# RETRACTION: Assessment of Toxicity and Beneficiary Effects of *Garcinia pedunculata* on the Hematological, Biochemical, and Histological Homeostasis in Rats

**DOI:** 10.1155/ecam/9795282

**Published:** 2026-07-31

**Authors:** Evidence-Based Complementary and Alternative Medicine

RETRACTION: S. Paul, M. Y. Ali, N.‐E.‐N. Rumpa, et al., “Assessment of Toxicity and Beneficiary Effects of *Garcinia pedunculata* on the Hematological, Biochemical, and Histological Homeostasis in Rats,” *Evidence-Based Complementary and Alternative Medicine*, 2017, 4686104, https://doi.org/10.1155/2017/4686104.

The above article, published online on 22 January 2017 in Wiley Online Library (https://wileyonlinelibrary.com), has been retracted by agreement by John Wiley & Sons Ltd. The retraction has been agreed due to concerns identified in Figure 6, some of which have been raised on PubPeer [1]. A summary of the concerns is as follows:

• In Figure 6, there is a large region of the C‐brain and Gp‐Brain panels which appears to overlap
• In Figure 6, there is a large region of the C‐spleen and Gp‐spleen panels which appears to overlap
• In Figure 6, there are unexpected, repeated regions between the C‐brain panel and the Pomelo‐brain panel in Figure 6 of [2], which shares some of the same authors
• In Figure 6, there are unexpected, repeated regions between the C‐stomach panel and the Pomelo‐stomach panel in Figure 6 of [2]
• In Figure 6, there are unexpected, repeated regions between the Gp‐brain panel and the C‐brain panel in Figure 6 of [2]
• In Figure 6, there are unexpected, repeated regions between the Gp‐brain panel and the Pomelo‐brain panel in Figure 6 of [2]
• In Figure 6, the Gp‐Heart panel appears identical to the Pomelo‐heart panel in Figure 6 of [2]
• In Figure 6, the C‐Heart panel appears similar to the C‐heart panel in Figure 6 of [2]

After assessment of the concerns and the author’s response, the results and conclusions can no longer be considered reliable, and the article is therefore retracted.

Sudip Paul agrees to the retraction; however, no response was received from the remaining authors.
